# Kv1.3 voltage-gated potassium channels link cellular respiration to proliferation through a non-conducting mechanism

**DOI:** 10.1038/s41419-021-03627-6

**Published:** 2021-04-07

**Authors:** Faye L. Styles, Moza M. Al-Owais, Jason L. Scragg, Eulashini Chuntharpursat-Bon, Nishani T. Hettiarachchi, Jonathan D. Lippiat, Aisling Minard, Robin S. Bon, Karen Porter, Piruthivi Sukumar, Chris Peers, Lee D. Roberts

**Affiliations:** 1 School of Medicine, University of Leeds, Leeds, LS2 9JT UK; 2 Faculty of Biological Sciences, University of Leeds, Leeds, LS2 9JT UK; 3 School of Chemistry, University of Leeds, Leeds, LS2 9JT UK

**Keywords:** Cell growth, Energy metabolism

## Abstract

Cellular energy metabolism is fundamental for all biological functions. Cellular proliferation requires extensive metabolic reprogramming and has a high energy demand. The Kv1.3 voltage-gated potassium channel drives cellular proliferation. Kv1.3 channels localise to mitochondria. Using high-resolution respirometry, we show Kv1.3 channels increase oxidative phosphorylation, independently of redox balance, mitochondrial membrane potential or calcium signalling. Kv1.3-induced respiration increased reactive oxygen species production. Reducing reactive oxygen concentrations inhibited Kv1.3-induced proliferation. Selective Kv1.3 mutation identified that channel-induced respiration required an intact voltage sensor and C-terminal ERK1/2 phosphorylation site, but is channel pore independent. We show Kv1.3 channels regulate respiration through a non-conducting mechanism to generate reactive oxygen species which drive proliferation. This study identifies a Kv1.3-mediated mechanism underlying the metabolic regulation of proliferation, which may provide a therapeutic target for diseases characterised by dysfunctional proliferation and cell growth.

## Introduction

Cellular energy metabolism is key to all biological processes. Biosynthesis of the macromolecular components of cells requires energy, generated in the mitochondria, in the form of ATP. Therefore redox regulation, and balancing the metabolic needs of oxidative phosphorylation (OXPHOS) required for ATP production and the generation of new lipids and nuclear material, is essential for cell proliferation. The mechanisms through which cells regulate their metabolism to drive cell growth and proliferation is not fully understood. These processes involve integrated environmental sensing and intracellular signalling, coupling the initiators of proliferation with the regulation of energy metabolism^1,2^. Here, we propose a role for a voltage-gated ion channel in the process linking proliferation and energy metabolism.

Voltage-gated potassium (Kv) channels are transmembrane proteins that facilitate K^+^ movement through membranes via their intrinsic pores. K^+^ influx into a cell or organelle activates signalling cascades that can regulate proliferation^3^, cell volume^4^, apoptosis, migration^5^ and energetics^6,7^. The Kv1.3 channel is present in a wide-range of tissues including the brain^8,9^, epithelial cells^10^, adipose tissue^6^ and both skeletal and smooth muscle cells^11,12^. Kv1.3 channels stimulate cellular proliferation^3,13–15^ with mechanisms implicated including ion conducting effects on plasma membrane potential^16^, Ca^2+^ influx^15^ and cell volume regulation^17^. Non-conducting signalling mechanisms, independent of ion influx/efflux, are emerging. These arise due to interactions between the channels and signalling/scaffolding proteins that either alter the channel’s cellular location or couple the channel to intracellular second messengers^18,19^.

Cellular proliferation has a high energy demand, requiring ATP for processes ranging from signal transduction to DNA, RNA and protein synthesis^20^. Besides the plasma membrane, Kv1.3 has been reported at intracellular sites, including the nucleus^21^ and the ER^22^. Plasma membrane and intracellular Kv1.3 channels may have distinct roles in cellular homoeostasis^18,23,24^. Kv1.3 has also been found in the inner mitochondrial membrane, the site of ATP synthesis^23,25,26^.

We hypothesised that the Kv1.3 channel may coordinate the energy demands required for Kv1.3-stimulated proliferation. We identify that intracellular Kv1.3 channels function through a non-conducting mechanism to regulate cellular energetics and demonstrate that regulation of respiration by the channel is a stimulatory mechanism for Kv1.3-mediated proliferation.

## Materials and Methods

### Cell culture and generation of Kv1.3 and Kv1.5 cells

Wild type (WT) HEK293 (ECACC, Public Health England, Porton Down, UK) and HEK293/Kv1.3 cells were cultured in DMEM supplemented with 10% (v/v) foetal bovine serum (BioSera, East Sussex, UK), 1% (v/v) Antibiotic-Antimycotic (10,000 units/mL penicillin, 10,000 μg/mL streptomycin, 25 μg/mL Fungizone™), 1% (v/v) non-essential amino acids, 0.1% (v/v) gentamicin (50 mg/ml), 1% (v/v) GlutaMAX and geneticin (G418 Sulfate, 600 ug/ml) (5% CO_2_; 37 °C). All cell culture reagents were purchased from Gibco-BRL (Paisley, UK) unless otherwise stated.

HEK293/Kv1.3 cells, and plasmids containing cDNA encoding mutant Kv1.3 channels were a gift from Professors José Ramón López López and Teresa Pérez-García (University of Valladolid). Kv1.3-P89 is a voltage sensitive, non-conducting channel containing a point mutation (W389F) in the S5-S6 linker of the Kv1.3-GFP fusion sequence (Supplementary Fig. S1A). Kv1.3-P93 is a non-conducting, voltage-insensitive channel and was generated as for Kv1.3-P89 but with three additional mutations (R320N/L321A/R326I) in the S4 (voltage sensor) region (Supplementary Fig. S1B). Kv1.3-P118 is a Kv1.3-mCherry fusion protein. Kv1.3-P121 is a phosphorylation-defective Kv1.3-mCherry fusion protein containing a point mutation (Y447A) in Kv1.3’s C-terminal region (Supplementary Fig. S1C)^13,18^. Plasmid maps are shown in Supplementary Fig. S2 (Supplementary Tables 1 and 2).

WT HEK293 cells were transfected with plasmid DNA (control HEK293) or plasmid DNA containing Kv1.3, or mutant Kv1.3 channels by nucleofection using Cell Line Nucleofector Kit V and Nucleofector™ Programme Q-01 (Lonza, Castleford, UK). Transfected cells were selected with G-418 antibiotic (1 mg/ml), 2 days after transfection. Selection was applied for 4 weeks, colonies picked, grown to confluence and screened electrophysiologically. Stable expression of mutant Kv1.3 channels was confirmed using immunoblotting (Supplementary Fig. S3).

Kv1.5 cDNA was amplified from a human foetal brain cDNA library (Clontech, UK) and HEK293/Kv1.5 cells were engineered to express the Kv1.5 channel (KCNA5) as described^27^.

### Proliferation assays

Proliferation assays followed established protocols^28–30^. Cells were seeded at 2 × 10^4^ cells per well of a 24-well plate. After 6 h media was replaced with serum-free media (SFM) overnight. On day 0, SFM was removed and growth media (1 ml) was added to each well. For pharmacological studies, ShK-Dap22 (1–200 pM), PAPTP (100 nM), PAP-1 (100 nM) or MitoQ (5 μM) were added on day 0. Cells were counted in triplicate on days 0, 1, 2 and 3 using a Bio-Rad TC10 cell counter (Bio-Rad Laboratories, USA).

### High-resolution respirometry

Respirometry (Oxygraph‐2K; Oroboros Instruments, Austria)^31^ was performed in intact HEK293 cells using established methods^32^. Briefly, 1 × 10^6^ cells/cm^3^ in SFM were added to the respiratory chambers and routine respiration measured. Non-phosphorylating leak was determined (2 μg/ml oligomycin). Maximum uncoupled electron transfer system (ETS) was measured using carbonyl cyanide-*p*-trifluoromethoxyphenylhydrazone (FCCP; 0.5 μM). Rotenone (2.5 μM) and Antimycin A (0.5 μM) were added to determine residual oxygen consumption (ROX). Complex IV activity was assessed using ascorbate (0.5 mM) and *N*, *N*, *N*’, *N*’-Tetramethyl-*p*-phenylenediamine dihydrochloride (2 mM). Sodium azide (20 mM) was added to inhibit mitochondrial respiration^33^.

### Citrate synthase assay

Citrate synthase assay followed described methods^34^.

### Immunocytochemistry

Cells were cultured on poly-l-lysine coated coverslips until 30–40% confluent. Control HEK293 and HEK293/Kv1.3 cells were fixed (4% paraformaldehyde, 20 min), washed (3 × 1 ml DPBS) and incubated in DPBS (0.05% Triton X100, 10% NGS; 20 min). Cells were washed (DPBS, 1% NGS) Anti-Kv1.3 Clone L23/27, mouse monoclonal (1:500; cat no. 75-009, NeuroMab, Antibodies Incorporated, UK) or Anti-ATPB rabbit polyclonal (1:500; cat no. ab128743, Abcam, UK) antibody were added in 1% NGS and incubated in a humidified chamber overnight at 4 ^o^C. Coverslips were washed with DPBS with 1% NGS (3 x 1ml). Alexa Fluor 488 Donkey anti-Mouse IgG (cat no. R37114, ThermoFisher) secondary antibody or HRP goat anti-rabbit IgG H + L secondary antibody (cat no. 926-80011 LI-COR Biosciences UK Ltd, Cambridge, UK) was added (1:2000 in 1% NGS) for 1 h. Cells were washed with DPBS with 1% NGS (3 × 1 ml), and mounted with VECTASHIELD Mounting Medium with DAPI (VECTOR Laboratories Ltd, UK). Mitochondria were imaged following incubation with MitoTracker Red CMXRos (ThermoFisher) (100 nM; 30 min; 37 °C; 5% CO_2_). Excitation/emission wavelengths: 579/599 nm. Imaging was perfomed using a Zeiss LSM800 microscope and processed using Zen Lite 64 (Zeiss, UK) and Image J.

### Cellular ROS assays

Cells were treated with 5 μM CellROX Deep Red (ThermoFisher Scientific, UK) (30 min; 37 °C; 5% CO_2_) with or without a 100 μM menadione 1 h pretreatment.

### Electrophysiology

Cells were perfused at 2–4 mL/min (volume 80 μL) with recording solution (NaCl 141 mM, HEPES 10 mM, KCl 4.7 mM, MgCl_2_ 1.2 mM, CaCl_2_ 1.8 mM, D-glucose 10 mM, pH 7.4, 21–24 °C) in a recording chamber mounted on an Olympus CK40 inverted microscope. Whole-cell patch-clamp recordings were made using patch pipettes (3–7 MΩ resistance) with electrode solution (EGTA 10 mM, HEPES 10 mM, KCl 125 mM, MgCl_2_ 4 mM and MgATP 5 mM, pH 7.2). Experiments were recorded on an Axopatch 200B amplifier (ALA Scientific Instruments, NY, USA), and stored and digitised using a Digidata 1322A (Axon, Molecular Devices, USA) and pClamp 10 (Molecular Devices), respectively. Two protocols were adopted: (1) Current-voltage (I/V) relationships measured by stepping from −80 mV holding potential to voltages between −60 and +60 mV in 10 mV increments for 300 ms and (2) a single step from −80 to +40 mV for 200 ms.

### Cairn photometry

Cells were cultured on poly-l-lysine coated coverslips until 90% confluent, washed (DPBS, 2 × 2 ml), treated with 20 nM tetramethylrhodamine, methyl ester (TMRM) (30 min, 5% CO_2_, 37 °C), washed (DPBS, 2 × 2 ml) and placed in Hank’s balanced salt solution. Coverslips were transferred to the recording chamber of an inverted epi-fluorescence microscope and perfused with recording solution (as for electrophysiology). Recordings were made using a Cairn Research ME-SE Photometry system (Cairn Research, UK). TMRM was excited by a Xeon Arc lamp (excitation/emission wavelengths: 548/574 nm).

### Flow cytometry

Ca^2+^ levels were measured using RHOD-2AM (20 nM) at 548/574 nm (excitation/emission wavelengths); mitochondrial membrane potential (MMP) was investigated using TMRM (1 μM) at 552/581 nm and NADH autofluorescence was measured at 350/470 nm. Gates were applied using unstained controls. Cells were analysed on a BD LSRFortessa™ analyser (BD Biosciences, USA) using the BD FACSDiva software (BD Biosciences, USA).

### Immunoblotting

Cells were lysed with M-per^TM^ (Perbio Science, UK) containing mini protease inhibitors (Roche Diagnostics UK, UK). Protein concentrations were determined using a BCA assay (Pierce, USA). Cell lysate was added to sample buffer (1:5; 60 mM Tris-Cl pH 6.8, 2% SDS, 10% glycerol, 5% β-mercaptoethanol, 0.01% bromophenol blue). Samples (10–20 μg protein) were loaded on to 12.5% polyacrylamide-SDS gels (Bio-Rad, UK) and separated at 200 V (45 min), then transferred to polyvinyl difluoride membranes (30 V, overnight at 4 °C; Millipore Corporation, Massachusetts, USA). Membranes were blocked with 5% non-fat milk protein in TBS-Tween (TBST, 0.05%; 1 h) and treated with antibodies: Anti-Kv1.3 clone L23/27 mouse monoclonal primary antibody (1:500; cat no. 75-009, NeuroMab, USA); Anti-COX IV mouse monoclonal primary antibody (1:500; cat no. 33985 Abcam, UK); Anti β-Actin Clone AC-15 mouse monoclonal primary antibody (1:600; cat no. A1978, Sigma Aldrich, UK); Anti-ATPB rabbit polyclonal antibody (1:500; cat no. 65378 Abcam, UK) diluted in 1% non-fat milk protein in TBST (3 h). Membranes were washed (6 × 5 min in TBST) and incubated (1 h) with HRP-conjugated goat anti-mouse IgG H + L secondary antibody for Anti-Kv1.3, Anti-COX IV and Anti β-Actin (1:20000; cat no. 926-80010, LI-COR Biosciences, UK) or HRP-conjugated goat anti-rabbit IgG H + L secondary antibody for Anti ATBP (1:20000; cat no. 926-80011, LI-COR Biosciences, UK). Membranes were washed in TBST (3 × 5 min), before annotation with a LI-COR WesternSure pen (LI-COR Biosciences, USA). A 1:1 mixture of LI-COR Western Sure ECL luminol enhancer solution with LI-COR Western Sure ECL stable peroxide was used to wash the membranes (5 min). Membranes were imaged on a C-Digit Blot Scanner (LI-COR Biosciences, UK) (12 min). Densitometry was quantified in Image J.

### Chemicals

PAPTP was synthesised according to literature^26^ and purified (assessed by 1H-NMR and 13C-NMR, Supplementary Fig. S4A, B).

### Statistical analysis

Sample sizes were calculated using power calculations. Where sample sizes were not calculated sample size was considered adequate based on the size and reproducibility of between group differences. Samples were randomly assigned to experimental groups. Group variance was analysed with an *F-*test. Data were analysed using either a paired, or unpaired two-tailed Student’s *t*-test and/or one-way and two-way ANOVA with a Sidak’s or Tukey’s post hoc test. Significance was determined when *p* ≤ 0.05.

## Results

### Kv1.3 expression increases cellular proliferation and respiration

**Fig. 1:**
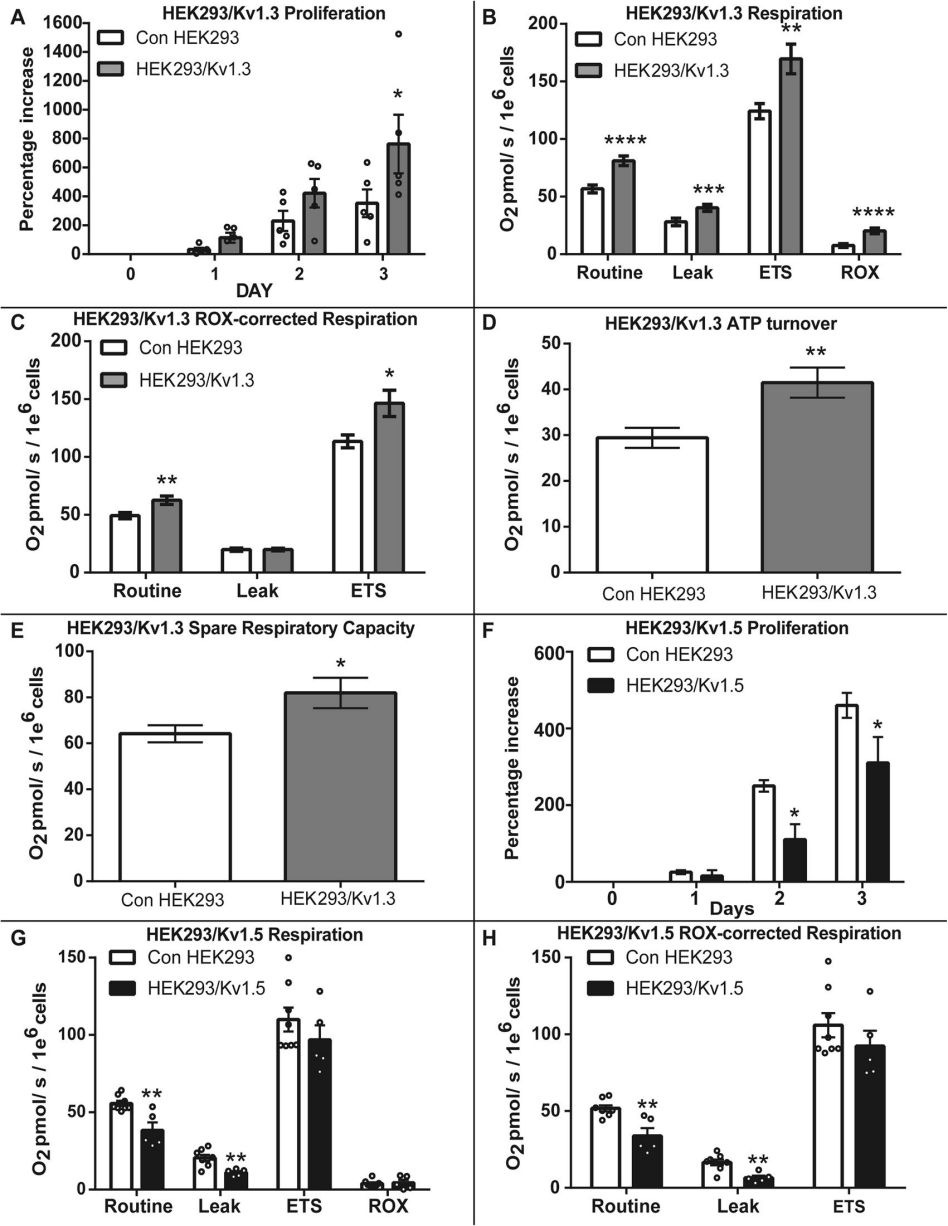
Kv1.3 expression increases cellular proliferation and respiration. **A** HEK293/Kv1.3 cells exhibit enhanced proliferation compared with control HEK293 cells. The percentage increase in cell number over 3 days normalised to day 0 (*n* = 5). **B** Routine, Leak, maximal electron transfer system (ETS) and residual (ROX) O_2_ consumption in HEK293/Kv1.3 (*n* = 38) and control HEK293 (*n* = 27) cells measured by high-resolution respirometry. **C** ROX-corrected Routine, Leak and ETS O_2_ consumption in HEK293/Kv1.3 (*n* = 38) and control HEK293 (*n* = 27) cells measured by high-resolution respirometry. **D** ATP turnover in HEK293/Kv1.3 (*n* = 38) cells and control HEK293 (*n* = 27) cells. **E** Spare respiratory capacity in HEK293/Kv1.3 (*n* = 38) and control HEK293 (*n* = 27) cells. **F** Proliferation is suppressed in HEK293/Kv1.5 cells (*n* = 12) compared with control HEK293 (*n* = 15). Percentage increase in cell number at day 1, 2 and 3 from day 0. **G** Routine, Leak, ETS and ROX O_2_ consumption in HEK293/Kv1.5 (*n* = 5) and control HEK293 (*n* = 8) cells measured by high-resolution respirometry. **H** ROX-corrected Routine, Leak, and ETS O_2_ consumption in HEK293/Kv1.5 (*n* = 5) and control HEK293 (*n* = 8) cells measured by high-resolution respirometry. Data expressed as the mean ± SEM. Data were analysed using either Student’s *t*-test or two-way ANOVA. *p < 0.05, **p < 0.01, ***p < 0.001 and ****p < 0.0001.

HEK293 cells were transfected to express the Kv1.3 channel. Immunocytochemistry immunoblotting and electrophysiology (Supplementary Fig. S5A–F) confirmed that the HEK293/Kv1.3 cells expressed functional Kv1.3. HEK293/Kv1.3 cells exhibited increased proliferation compared to control HEK293 cells (Fig. 1A).

High-resolution respirometry identified that Kv1.3 expression increased mitochondrial respiration in HEK293 cells (Fig. 1B). Kv1.3 expression increased Routine, non-phosphorylating proton Leak, maximal uncoupled ETS and non-OXPHOS ROX respiration. When corrected for ROX, Routine and maximal respiration remained elevated in Kv1.3-expressing cells (Fig. 1C). The ATP turnover (Fig. 1D) and spare respiratory capacity (Fig. 1E) were increased by Kv1.3 expression.

In contrast to Kv1.3, expression of the voltage-gated K^+^ channel Kv1.5 reduces proliferation^35^. Respirometry was used to determine whether Kv1.5 also has contrasting effects on respiration in HEK293 cells expressing Kv1.5. HEK293/Kv1.5 cells exhibited reduced proliferation (Fig. 1F) and had lower Routine/basal and Leak respiration compared to control HEK293 or HEK293Kv1.3 cells, independent of ROX (Fig. 1G, H). The opposing effects on proliferation by Kv1.3 and Kv1.5 channel expression are mirrored by effects on mitochondrial respiration.

### Plasma membrane Kv1.3 channel K^+^ transport does not regulate proliferation or respiration

**Fig. 2:**
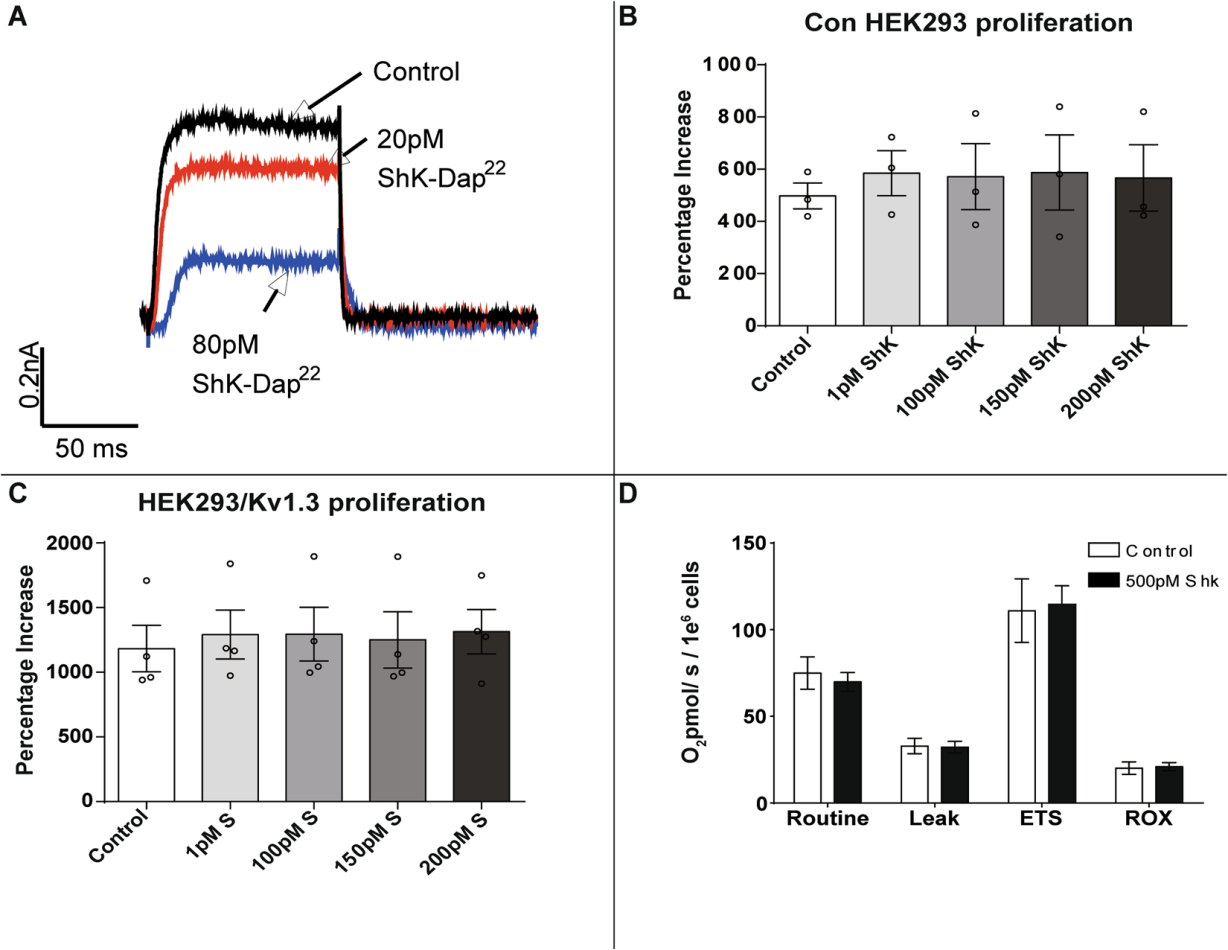
Plasma membrane Kv1.3 channels do not regulate proliferation or respiration. **A** Whole cell voltage clamp trace showing inhibition of Kv1.3 currents by ShK-Dap22 (20 and 80 pM). **B** Proliferation of control HEK293 cells treated with ShK-Dap22 (1–200 pM). The percentage increase in cell number over 3 days normalised to day 0 (*n* = 3). **C** Proliferation of HEK293/Kv1.3 cells treated with ShK-Dap22 (1–200 pM). The percentage increase in cell number over 3 days normalised to day 0 (*n* = 4). **D** Routine, Leak, maximal electron transfer system (ETS) and residual (ROX) O_2_ consumption in HEK293/Kv1.3 cells treated with 500 pM ShK-Dap22 (*n* = 10). Data expressed as mean ± SEM.

Next we determined whether K^+^ transport by Kv1.3 channels in the plasma membrane regulated the increase in cellular proliferation and respiration. ShK-Dap22 is a potent, membrane impermeable, Kv1.3 inhibitor^36^. Electrophysiology recordings confirmed that ShK-Dap22 inhibited plasma membrane Kv1.3 in HEK293/Kv1.3 cells (Fig. 2A). The IC_50_ of ShK-Dap22 for Kv1.3 current inhibition is 39.6 pM^36^. ShK-Dap22 (80 pM) reduced the potassium currents in HEK293/Kv1.3 cells. A concentration-response was used to assess the effect of ShK-Dap22 (1–200 pM) on proliferation of control HEK293 (Fig. 2B) and HEK293/Kv1.3 cells (Fig. 2C). ShK-Dap22 did not affect proliferation. K^+^ transport through plasma membrane Kv1.3 does not induce channel-dependent proliferation.

We then assessed whether plasma membrane Kv1.3 channels induce mitochondrial respiration. HEK293/Kv1.3 cells were treated with ShK-Dap22 and analysed with respirometry. ShK-Dap22 up to 500 pM had no effect on Routine, Leak, ETS or ROX respiration in HEK293/Kv1.3 cells (Fig. 2D). Plasma membrane Kv1.3 channel conductance does not induce channel-dependent mitochondrial respiration.

### Kv1.3 channels localise to the mitochondria but do not increase cellular mitochondrial content

**Fig. 3:**
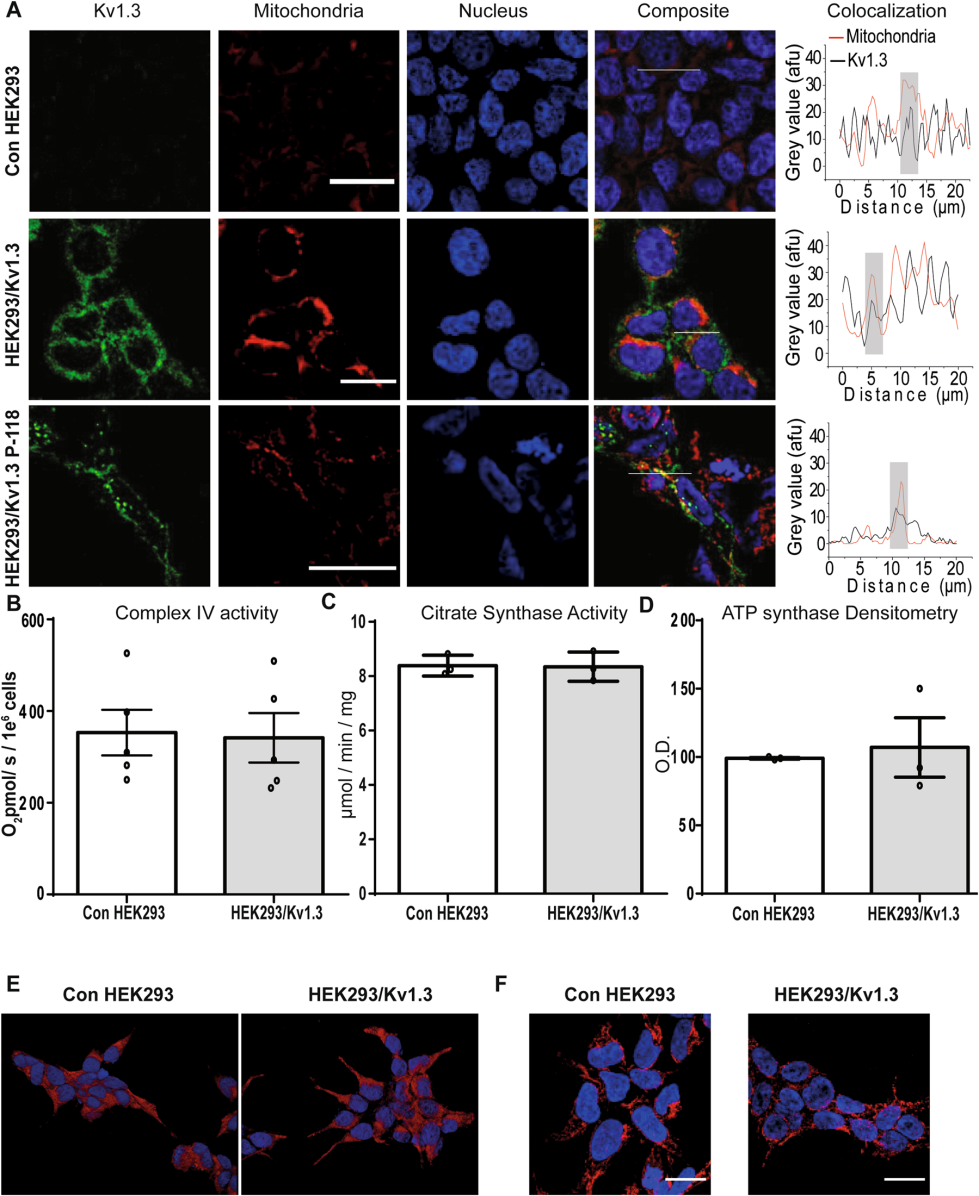
Kv1.3 channels localise to the mitochondria. **A** Control HEK293 and HEK293Kv1.3 cells stained with Kv1.3 monoclonal primary antibody (green), MitoTracker Red CMXRos (red) and DAPI (blue). HEK293Kv1.3-mCherry cells with Kv1.3-mCherry (green) and immunostaining of mitochondrial cytochrome C oxidase (red). The white line on the merge images correspond to the plotted line profiles. The plotted line profile show the intensity at each point along the line. Scale bars are 25 μm. **B** Complex IV respiratory assay in HEK293/Kv1.3 and control HEK293 cells (*n* = 5). **C** Citrate synthase activity assay in control HEK293 and HEK293/Kv1.3 cells (*n* = 3). **D** Optical density (O.D.) from Western blotting of mitochondrial ATP synthase protein in control HEK293 and HEK293/Kv1.3 cells (*n* = 3). **E** Maximum intensity images of control HEK293 (left column) and HEK293/Kv1.3 cells (right column) pretreated with 100 nM MitoTracker Red CMXRos (red). Cell nuclei are stained with DAPI (blue). Scale bars represent 50 μm. **F** Maximum intensity images of control HEK293 (left column) and HEK293/Kv1.3 cells (right column) stained with anti-Complex IV antibody (red). Cell nuclei are stained with DAPI (blue). Scale bars represent 25 μm. Data were mean ± SEM.

The Kv1.3-induced increase in mitochondrial respiration was not activated by K+ transport through plasma membrane Kv1.3 channels. Kv1.3 has been reported to localise to the mitochondrial membrane^23,25,26^. We investigated whether Kv1.3 channels localise to the mitochondria in our model. Immunocytochemistry was employed to investigate Kv1.3 cellular localisation in control HEK293 and HEK293/Kv1.3 cells. Cells were stained for Kv1.3 (anti-Kv1.3 antibody and fluorescent secondary reporter; green), and mitochondria (MitoTracker CMXRos; red) and imaged using confocal microscopy (Fig. 3A). HEK293 cells do not exhibit autofluorescence within the MitoTracker detection wavelengths in cells not stained with MitoTracker (Supplementary Fig. S6A, B). As a secondary approach, HEK293 cells expressing mCherry-tagged Kv1.3 channels (HEK293/Kv1.3-P118; green) were immunofluorescently stained for mitochondrial complex IV (red) (Fig. 3A). In both cases Kv1.3 and mitochondria co-localisation was visualised in yellow and observed using a mitochondrial and Kv1.3 florescence intensity plotted line profile through a cross section of the images. HEK293/Kv1.3 cells had greater Kv1.3 staining. The Kv1.3 channels were observed to co-localise with mitochondria. Interestingly, Kv1.3 expression is not ubiquitously distributed among mitochondria. Consistent with findings in the literature, our data suggest that the majority of the Kv1.3 channel protein is intracellular^13^.

Next we investigated whether increased respiration in Kv1.3-expressing cells was driven by increased mitochondrial content. Canonical biochemical markers of mitochondrial content, complex IV activity using respirometry (Fig. 3B) and citrate synthase activity (Fig. 3C) were determined in control HEK293 and HEK293/Kv1.3 cells. Expression of the mitochondrial protein ATP synthase was also examined using immunoblotting in HEK293/Kv.13 cells (Fig. 3D and Supplementary Fig. S6C). Kv1.3 expression did not increase cellular mitochondrial content.

We reasoned that Kv1.3-induced increases in mitochondrial respiration may occur via alterations to the mitochondrial network. Confocal microscopy was used to investigate the mitochondrial network of HEK293/Kv1.3 cells. Cells were either stained with Mitotracker (red) (Fig. 3E) or immunostained for mitochondrial complex IV (red) (Fig. 3F). The gross structure of the mitochondrial network was unchanged by Kv1.3 expression. Kv1.3 does not exert its effects on cellular respiration through mitochondrial biogenesis or network reorganisation.

### Intracellular Kv1.3 channels regulate proliferation and respiration

**Fig. 4:**
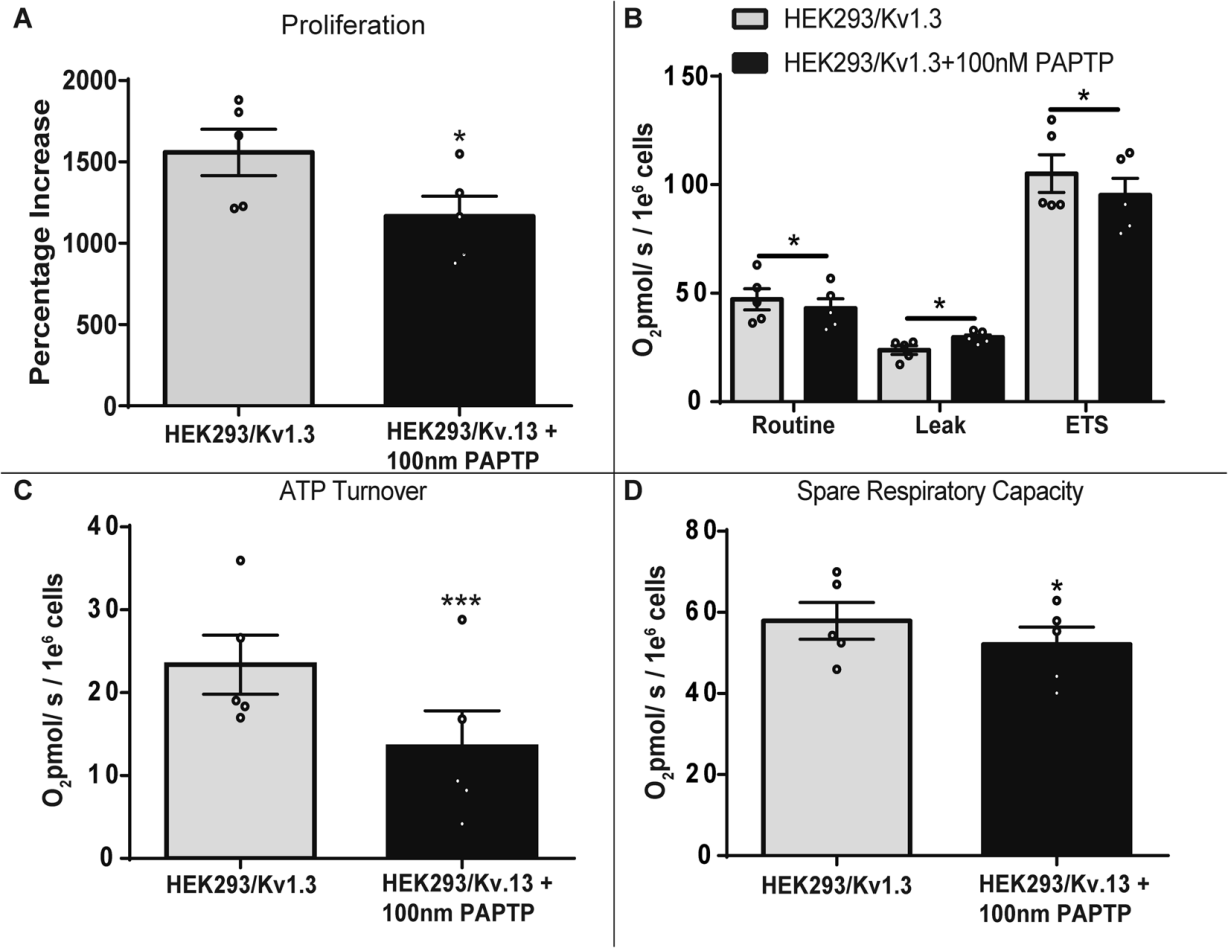
Intracellular Kv1.3 channels regulate proliferation and respiration. **A** Proliferation assay of HEK293/Kv1.3 cells treated with PAPTP (100 nM; *n* = 5). **B** Residual O_2_ consumption (ROX) corrected Routine, Leak and maximal ETS respiration in HEK293/Kv1.3 cells treated with PAPTP (100 nM; *n* = 5). **C** ATP turnover in HEK293/Kv1.3 cells treated with PAPTP (100 nM; *n* = 5). **D** Spare respiratory capacity in HEK293/Kv1.3 cells treated with PAPTP (100 nM; *n* = 5). Data were mean ± SEM. Data were analysed using a paired Student’s *t*-test. *p < 0.05 and ***p < 0.001.

Plasma membrane Kv1.3 ion conductance did not regulate Kv1.3-induced respiration. We investigated the role of intracellular Kv1.3 channels in the regulation of proliferation and respiration. PAP-1 is a membrane permeable inhibitor of intracellular Kv1.3 channels, including those located at the *cis*-Golgi, ER, nucleus and mitochondria^21,22^. PAP-1 functions by stabilising Kv1.3’s inactivate state^21,22^. PAP-1 can be bound to triphenyl phosphonium lipophilic cation, forming the Kv1.3 inhibitor PAPTP, which inhibits the Kv1.3 whole-cell current and is pharmacologically targeted to the mitochondria^26,37,38^. PAPTP was used to probe the role of the intracellular ion channels in proliferation and mitochondrial respiration. PAPTP (100 nM) significantly reduced HEK293/Kv1.3 cell proliferation (Fig. 4A). The observed decrease in proliferation was not due to PAPTP cytotoxicity (Supplementary Fig. S7). We compared the effect of the mitochondrially-targeted Kv1.3 inhibitor PAPTP (100 nM) on HEK293/Kv1.3 cell proliferation to its non-specific intracellular equivalent PAP-1 (100 nM) (Supplementary Fig. S8). The mitochondrially-targeted compound PAPTP had a greater inhibitory effect on HEK293/Kv1.3 proliferation.

The effect of mitochondrially-targeted Kv1.3 channel inhibition on respiration rate was examined in HEK293/Kv1.3 cells using PAPTP (Fig. 4B). PAPTP reduced Routine and maximal ETS respiration, whilst increasing proton Leak respiration. PAPTP also reduced both ATP turnover rate and spare respiratory capacity in HEK293/Kv1.3 cells (Fig. 4C, D). PAPTP did not affect proliferation, or Routine, maximal ETS and Leak respiration in control HEK293 cells (Supplementary Fig. S9), indicating that PAPTP’s effects in HEK293/Kv1.3 cells are not due to off-target activity. These data suggest that mitochondrial Kv1.3 channels may regulate proliferation and respiration.

### Reactive oxygen species generation links Kv1.3-induced respiration to cellular proliferation

**Fig. 5:**
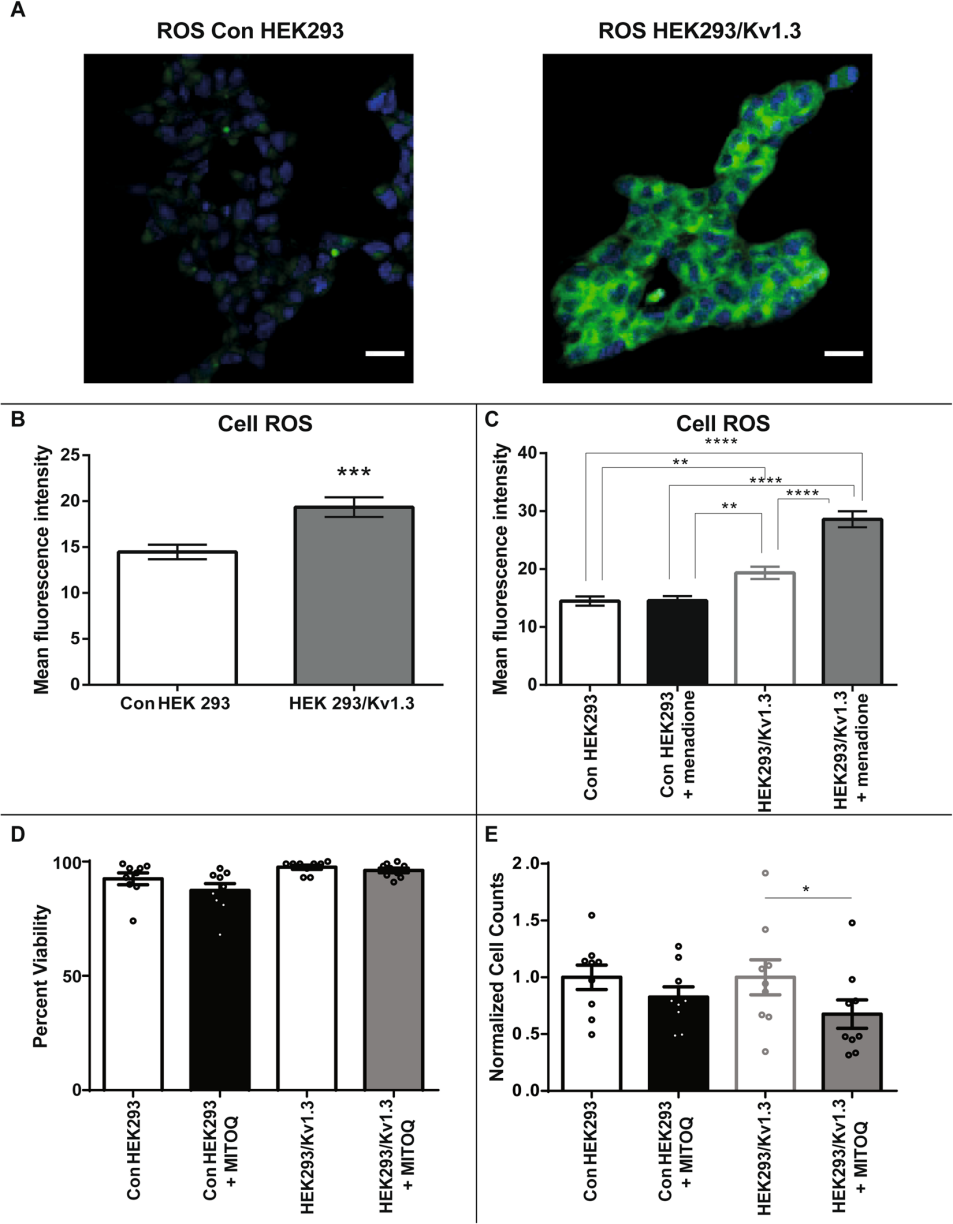
Reactive oxygen species link mitochondrial Kv1.3-induced respiration to cellular proliferation. **A** Representative images showing cellular reactive oxygen species (ROS) in control HEK293 (left) and HEK293/Kv1.3 (right) cells stained with 5 μM CellROX deep red reagent (green) and DAPI to highlight cell nuclei (blue). Scale bars represent 25 μm. **B** ROS levels in control HEK293 cells and HEK293/Kv1.3 cells stained with 5 μM CellROX deep red reagent (*n* = 30). **C** ROS levels in control HEK293 and HEK293/Kv1.3 cells stained with 5 μM CellROX deep red reagent with and without 100 μM menadione pretreatment (*n* = 30). **D** Percentage viability of control HEK293 and HEK293/Kv1.3 cells with and without 5 μM MitoQ treatment assessed using trypan blue staining (*n* = 9). **E** Control HEK293 and HEK293/Kv1.3 cell counts following 3 days of proliferation, normalised to control, with and without 5 μM MitoQ (*n* = 9). Data were expressed as mean ± SEM and was analysed using either a Student’s *t*-test or two-Way ANOVA with Tukey’s post hoc test, *p < 0.05, **p < 0.01, ***p < 0.001 and ****p < 0.0001.

Mitochondria are the primary site of cellular reactive oxygen species (ROS) production. Kv1.3 expression increases mitochondrial respiration. HEK293/Kv1.3 cells displayed greater ROS concentrations than control HEK293 cells, determined using the cell permeable fluorescent ROS dye CellROX Deep Red (Fig. 5A, B). Menadione induces cellular ROS generation through redox cycling. ROS production in response to 100 μM menadione was greater in HEK293/Kv1.3 cells than in control HEK293 cells (Fig. 5C). These data suggest that increased mitochondrial respiration in HEK293/Kv1.3 cells leads to a greater capacity for ROS production. ROS can enhance cellular proliferation^39^. We investigated whether mitochondrial ROS in HEK293/Kv1.3 cells was driving the proliferative phenotype. MitoQ, a mitochondrially-targeted ROS scavenger, had no effect on cell viability (Fig. 5D), and significantly reduced proliferation in HEK293/Kv1.3 cells without affecting proliferation in control HEK293 cells (Fig. 5E). The increased respiration in Kv1.3 expressing cells increases capacity for ROS generation, which in turn drives the proliferative phenotype.

### Kv1.3 regulates respiration independently of mitochondrial membrane potential, Ca^2+^ or NADH redox status

We explored the mechanisms through which Kv1.3 regulates respiration. Mitochondrial ion channels can regulate the MMP to control respiration^40^. The MMP of HEK293/Kv1.3 and control HEK293 cells was investigated using the Cairn Photometry system. TMRM is a cell-permeant dye attracted to the negative charge of the mitochondrial membrane. TMRM accumulation is greater in HEK293/Kv1.3 cells, indicating greater MMP hyperpolarisation (Supplementary Fig. S10A). HEK293/Kv1.3 and control HEK293 cells were sorted by flow cytometry following either treatment with TMRM, or TMRM and the OXPHOS uncoupler FCCP. Flow cytometry confirmed Kv1.3-induced increases in HEK293 cells MMP (Supplementary Fig. S10B). FCCP dissipates the MMP releasing mitochondrial TMRM. There was a greater difference in the mean fluorescent intensity between TMRM and TMRM with FCCP treated HEK293/Kv1.3 cells compared with control HEK293 cells, indicating a more hyperpolarised MMP (Supplementary Fig. S10C). To investigate the role of Kv1.3 channels in the MMP phenotype, the mitochondrially-targeted Kv1.3 inhibitor PAPTP was added during TMRM incubation in both HEK293/Kv1.3 and control HEK293 cells. PAPTP did not affect the MMP, suggesting that the increased MMP observed in HEK293/Kv1.3 cells is not directly linked to mitochondrial Kv1.3 or the proliferative phenotype (Supplementary Fig. S10D).

Increased mitochondrial Ca^2+^ concentrations can drive dehydrogenase enzyme activity enhancing NADH availability (the principal OXPHOS electron donor) and increasing respiration. Both Ca^2+^ and NADH were measured in control HEK293 and HEK293/Kv1.3 cells as an indicator of mitochondrial redox status^41^. Cells were stained with the fluorescent Ca^2+^ indicator Rhod2-AM and analysed by flow cytometry. Cellular and mitochondrial Ca^2+^ levels were examined under two conditions; baseline Rhod2 fluorescence and Rhod2 fluorescence following addition of FCCP (20 µM). The mitochondrial Ca^2+^ range can be estimated by examining the difference in Rhod2 fluorescence between baseline and FCCP treatment. Kv1.3 expression did not affect cellular Ca^2+^ concentrations (Supplementary Fig. S10E) or mean mitochondrial Ca^2+^ range in HEK293 cells (Supplementary Fig. S10F).

NADH concentrations measured by autofluorescence detection using flow cytometry were greater in HEK293/Kv1.3 compared with control HEK293 cells (Supplementary Fig S10G). NADH autofluorescence was measured following 20 μM FCCP treatment to maximally oxidise the NAD^+^/NADH pool, and both 0.5 µM rotenone plus 2.5 µM Antimycin A treatment to maximally reduce the NAD^+^/NADH pool (Supplementary Fig. S10G). NADH concentrations were higher in HEK293/Kv1.3 cells compared with control HEK293 cells in all conditions. The difference in fluorescence between the maximally oxidised and maximally reduced NAD^+^/NADH pools (NADH range) estimates total mitochondrial NADH and NAD^+^^42^. HEK293/Kv1.3 cells had an increased NADH autofluorescence range compared to control HEK293 cells (Supplementary Fig. S10H). HEK293/Kv1.3 cells were treated with 100 nM PAPTP to inhibit Kv1.3 channels. PAPTP had no effect on NADH autofluorescence in HEK293/Kv1.3 cells (Supplementary Fig. S10I), suggesting that Kv1.3-induced increases in NADH do not mediate channel-enhanced respiration.

### Kv1.3 channels regulate proliferation and respiration via a non-conducting mechanism

We interrogated the structural and functional properties of Kv1.3 that may regulate respiration. HEK293 cells were transfected to express mutant Kv1.3. Kv1.3-P89 is a voltage sensitive but non-conducting Kv1.3 channel with intact gating properties. It has a single point mutation (W389F) in the channel pore region (S5-S6)^13^. Kv1.3-P93 is a voltage insensitive and non-conducting Kv1.3 channel which shares the Kv1.3-P89 (W389F) point mutation and has three additional point mutations in the voltage sensor (S4)^13^. This triple mutation (R320N, L321A and R326I) shifts the channel activation to potentials below −170 mV. The channel is inactive at physiological voltages and functions as an inward rectifier^43^. Kv1.3 phosphorylation by intracellular kinases is a common post-translational modification^44,45^. Kv1.3-P121 is voltage sensitive and conductance competent. However Kv1.3-P121 is phosphorylation-defective due to a Y447A substitution in its C-terminus which prevents phosphorylation of this site by the extracellular signal-regulated kinases 1/2 (ERK1/2)^13^.

**Fig. 6:**
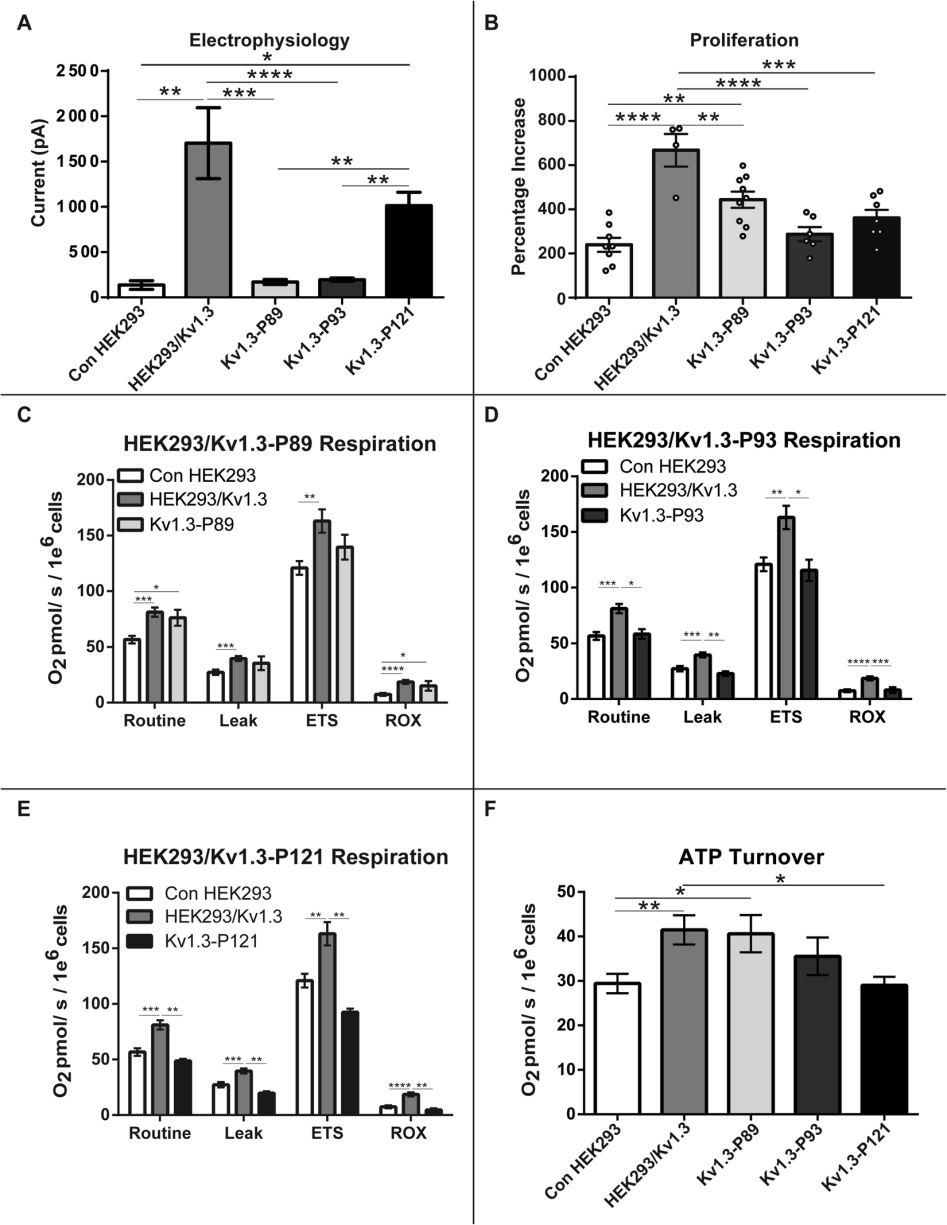
Mitochondrial Kv1.3 channels regulate proliferation and respiration via a non-conducting mechanism. **A** Electrophysiology currents for control HEK293, HEK293/Kv1.3, HEK/Kv1.3-P89, HEK/Kv1.3-P93 and HEK293/Kv1.3-P121 when depolarised from −80 mV to +40 mV (control HEK293 cells, *n* = 6; HEK293/Kv1.3 cells, *n* = 14; HEK293/Kv1.3-P89 cells, *n* = 9; HEK293/Kv1.3-P93 cells, *n* = 15; HEK293/Kv1.3-P121 cells, *n* = 23). **B** Proliferation of control HEK293, HEK293/Kv1.3, HEK293/Kv1.3-P89, HEK293/Kv1.3-P93 and HEK293/Kv1.3-P121 cells expressed as a percentage increase in cell number over 3 days (control HEK293 cells, *n* = 8; HEK293/Kv1.3 cells, *n* = 4; HEK293/Kv1.3-P89 cells, *n* = 9; HEK293/Kv1.3-P93 cells, *n* = 6; HEK293/Kv1.3-P121 cells, *n* = 7). **C** Routine, Leak, maximal electron transfer system (ETS) and residual (ROX) O_2_ consumption in control HEK293, HEK293/Kv1.3 and HEK293/Kv1.3-P89 cells (control HEK293, *n* = 27; HEK293/Kv1.3, *n* = 38; HEK293/Kv1.3-P89, *n* = 9). **D** Routine, Leak, maximal ETS and ROX O_2_ consumption in control HEK293, HEK293/Kv1.3 and HEK293/Kv1.3-P93 cells (control HEK293, *n* = 27; HEK293/Kv1.3, *n* = 38; HEK293/Kv1.3-P93, *n* = 9). **E** Routine, Leak, maximal ETS and ROX O_2_ consumption in control HEK293, HEK293/Kv1.3 and HEK293/Kv1.3-P121 cells (control HEK293, *n* = 27; HEK293/Kv1.3, *n* = 38; HEK293/Kv1.3-P121, *n* = 6). **F** ATP turnover in control HEK293, HEK293/Kv1.3, HEK293/Kv1.3-P89, HEK293/Kv1.3-P93 and HEK293/Kv1.3-P121 cells (control HEK293 *n* = 27; HEK293/Kv1.3 *n* = 38; HEK293/Kv1.3-P89 *n* = 9; HEK293/Kv1.3-P93 *n* = 9; HEK293/Kv1.3-P121 *n* = 6). Data were mean ± SEM. Data were analysed using one-way ANOVA with Tukeys post hoc test, *p < 0.05, **p < 0.01, ***p < 0.001 and ****p < 0.0001.

Using electrophysiology we confirmed that cells transfected with mutant Kv1.3 channels HEK293/Kv1.3-P89 and HEK293/Kv1.3-P93 are conductance deficient (Fig. 6A). HEK293/Kv1.3-P121 cells were conductance competent and exhibited comparable currents to HEK293/Kv1.3 cells (Fig. 6A).

We then determined the mechanism through which Kv1.3 channels regulate proliferation (Fig. 6B). Cells expressing Kv1.3 with a non-functioning pore (Kv1.3-P89) displayed increased proliferation when compared to control HEK293 cells. This suggests that ion conductance through the Kv1.3 channel is not essential for Kv1.3-induced proliferation. Disruption of the Kv1.3 voltage sensor (Kv1.3-P93) ablated the channels effect on proliferation. HEK293/Kv1.3-P121 cells also did not exhibit increased proliferation compared to control HEK293 cells. These data indicate that Kv1.3-induced proliferation is regulated via a non-conducting mechanism requiring both an intact voltage sensor and the C-terminal ERK1/2 phosphorylation site.

We assessed whether Kv1.3 channels also regulate respiration via the non-conducting mechanism. Cells transfected with Kv1.3-P89, Kv1.3-P93 and Kv1.3-P121 were analysed with respirometry and compared to control HEK293 and HEK293/Kv1.3 cells. Expression of Kv1.3 increased cellular respiration (Fig. 6C). The Routine, Leak, ETS and ROX respiration of HEK293/Kv1.3-P89 cells was not significantly different from that of HEK293/Kv1.3 cells (Fig. 6C), suggesting that Kv1.3-mediated K^+^ conductance is not necessary for Kv1.3-induced respiration. HEK293/Kv1.3-P93 cell Routine, Leak, ETS and ROX respiration was no different to control and reduced when compared to HEK293/Kv1.3 cells (Fig. 6D). These data are consistent with the observed reduction in proliferation in HEK293/Kv1.3-P93 cells compared with HEK293/Kv1.3 cells and suggest that the Kv1.3 voltage sensor is required for channel-induced respiration. Consistent with a reduced rate of proliferation, Routine, Leak, ETS and ROX respiration in HEK293/Kv1.3-P121 cells was also reduced compared to HEK293/Kv1.3 cells (Fig. 6E), suggesting that preservation of the Y447 ERK1/2 phosphorylation site is essential for Kv1.3-induced respiration.

Cellular proliferation has a high energy demand, requiring increased ATP turnover. ATP turnover rate was calculated for control, Kv1.3, Kv1.3-P121, Kv1.3-P89 and Kv1.3-P93 HEK293 cells (Fig. 6F). Disruption of the channel pore (HEK293/Kv1.3-P89) had no effect on Kv1.3-enhanced ATP turnover. However, there was no difference between the ATP turnover in control HEK293 cells, HEK293/Kv1.3-P93 and HEK293/Kv1.3-P121 cells. Loss of a functioning voltage sensor (Kv1.3-P93) and the ERK1/2 phosphorylation site (Kv1.3-P121) ablates Kv1.3-mediated enhanced ATP turnover.

## Discussion

The Kv1.3 channel enhances cellular proliferation and has been implicated in diseases of pathological proliferation including cancer and cardiovascular disease^26,46^. Our data suggests Kv1.3 located in the mitochondria may be important to this phenotype. Proliferation is a highly energy demanding process requiring increases in ATP turnover. Kv1.3 channels induced an increase in cellular respiration and ATP turnover, independent of effects on mitochondrial content. However, Kv1.3 expression was not ubiquitously distributed among mitochondria. Whether the ion channel is enriched in a specific mitochondrial subpopulation remains to be determined.

**Fig. 7:**
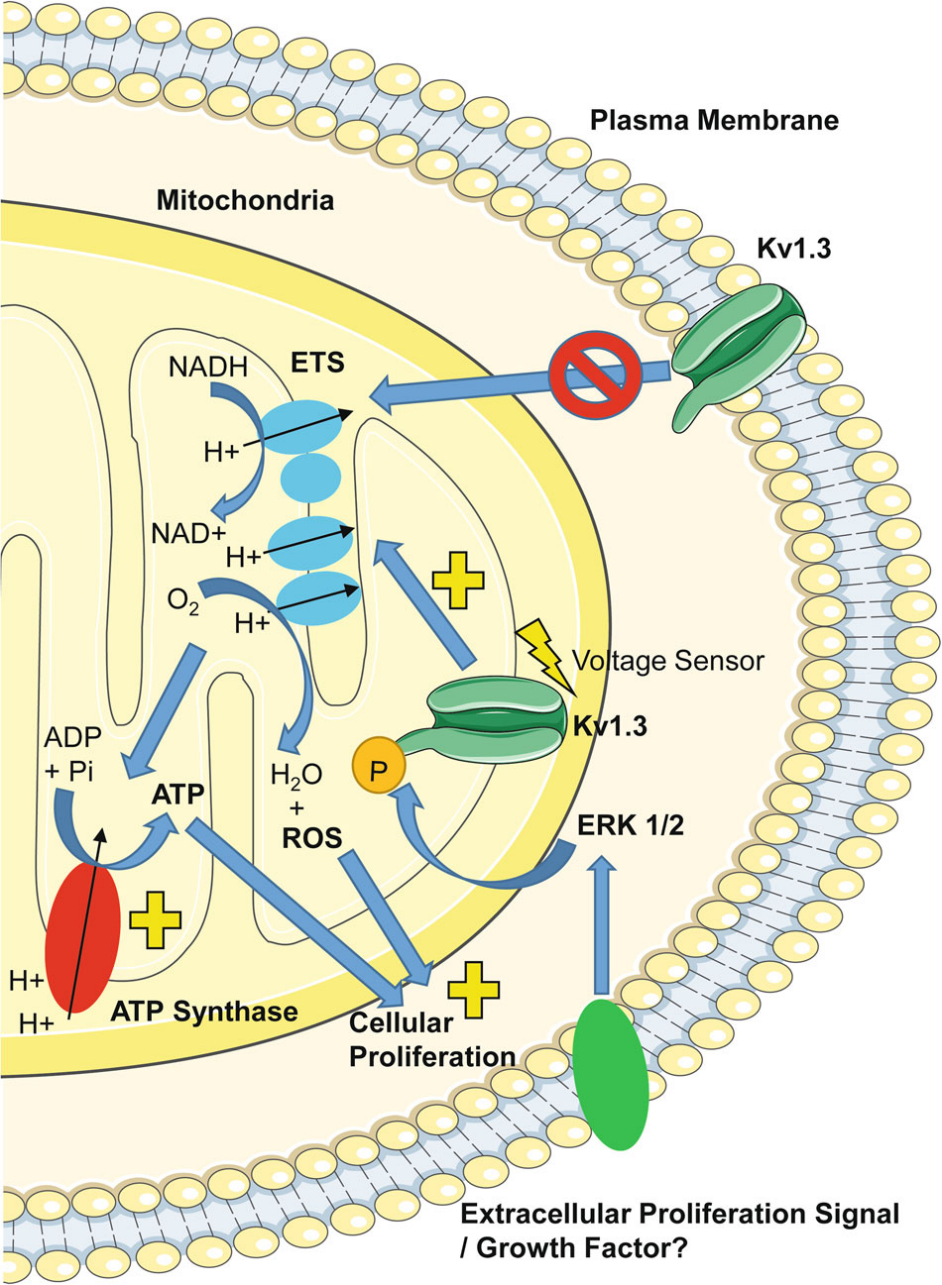
Diagrammatic representation of the proposed mechanism for Kv1.3-induced respiration and proliferation. Kv1.3 channels potentially located at the mitochondria require voltage sensing and an intact ERK1/2 phosphorylation site (Y447) to stimulate mitochondrial oxidative phosphorylation. Kv1.3-induced oxidative phosphorylation drives increased ATP turnover, meeting the increased energy demands needed for proliferation. Simultaneously, Kv1.3-induced oxidative phosphorylation generates mitochondrial reactive oxygen species (ROS) which stimulate the proliferative phenotype of the cells. This process is independent of plasma membrane Kv1.3 channel ion conductance. However, it may be speculated that growth factor receptors at the cell plasma membrane may signal to Kv1.3 channels via downstream ERK1/2-mediated phosphorylation.

Kv1.3-induced respiration had a direct mechanistic link to the proliferative phenotype beyond ATP generation. Kv1.3-driven OXPHOS increased the production of ROS which were required for proliferation. Using mutant Kv1.3 channels, we identified that Kv1.3-induced proliferation and respiration was independent of the channel pore. Mutation of the channel’s voltage sensor ablated Kv1.3’s effects on respiration and proliferation. Therefore the non-conducting process required to drive respiration remained linked to the channels’ ability to sense the extracellular potential. The activity of intracellular ion channels can be regulated by kinase-mediated signalling cascades^13,44,45^. Several pro-proliferative growth factors, including platelet-derived growth factor, signal via ERK to induce their cellular effects^35^. Kv1.3 has been identified as an ERK1/2 substrate with a tyrosine 447 phosphorylation site in the C-terminal region^13^. ERK signalling is required for Kv1.3-mediated proliferation^13^. Mutation of the phosphorylation site ablated the Kv1.3-induced increase in respiration and proliferation. This study proposes a mechanism for Kv1.3-mediated cellular proliferation whereby Kv1.3 channels require phosphorylation by ERK1/2 and the capacity to sense the extracellular potential to increase respiration, driving ROS production and, subsequently, proliferation (Fig. 7). The reliance on both voltage sensing and ERK phosphorylation for the regulation of respiration is consistent with a model in which, upon voltage sensing, Kv1.3 undergoes a conformational change exposing the ERK phosphorylation site in the C-terminus^3,13,47^. This is also consistent with the observation that PAPTP, which locks the channel in the inactive state, inhibits Kv1.3-induced respiration and proliferation. Kv1.3-induced respiration is not dependent on K^+^ ion conductance; therefore PAPTPs inhibitory effects may instead relate to preventing the conformational change in Kv1.3 that exposes the ERK phosphorylation site at residue 447.

We acknowledge limitations to our study. Our data using the mitochondrially-targeted Kv1.3 inhibitor PAPTP suggest that mitochondrial Kv1.3 channels may be important to the Kv1.3-induced proliferative and respiration phenotype. However, future studies confirming this association and linking the Kv1.3 organelle location to the non-conducting mechanism, potentially through mitochondrially-targeted versions of the channel pore, voltage sensor and ERK phosphorylation-defective Kv1.3 channels will be important.

We identify that Kv1.3 coordinates cellular respiration to meet the energy requirements needed for proliferation. We suggest a mechanism linking ERK1/2 signalling to Kv1.3-mediated increased respiration, ROS generation and proliferation. Our study has inferences for growth factor-mediated mechanisms that converge on ERK to drive physiological and pathological proliferation.

## Supplementary information

Supplementary Material

## Data Availability

All data and materials are available from the corresponding author on reasonable request.
