# Supplementary material for: Kv1.3 voltage-gated potassium channels link cellular respiration to proliferation through a non-conducting mechanism
Source: Cell Death Dis. 2021 Apr 7;12(4):372. doi: 10.1038/s41419-021-03627-6 (PMC8027666; doi:10.1038/s41419-021-03627-6)
Supplement: Supplementary file 1 — Supplementary Material [file 41419_2021_3627_MOESM1_ESM.docx]

**Supplementary Information**

**Kv1.3 voltage-gated potassium channels link cellular respiration to proliferation through a non-conducting mechanism**

Faye L Styles^1^, Moza M. Al-Owais^2^, Jason L Scragg^1^, Eulashini Chuntharpursat-Bon^1^,­ Nishani T Hettiarachchi^1^, Jonathan D. Lippiat^2^, Aisling Minard^3^ Robin S Bon^1^, Karen Porter^1^, Piruthivi Sukumar^1^, Chris Peers^1^, Lee D Roberts^1^

1. School of Medicine, University of Leeds, Leeds LS2 9JT, UK.

2. Faculty of Biological Sciences, University of Leeds, Leeds, LS2 9JT, UK.

3. School of Chemistry, University of Leeds, Leeds LS2 9JT, UK.


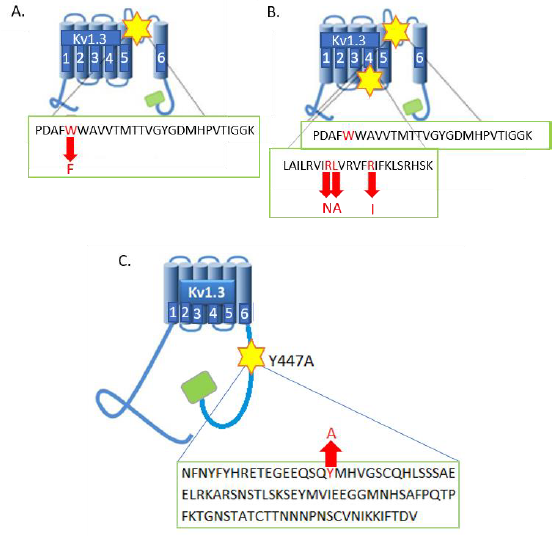


**C**

**B**

**A**

**Supplementary Figure 1. Schematic showing the location of the mutations in the Kv1.3 channels. A**) Single point mutation (W389F) in S5-S6 linker of the Kv1.3-P89 channel. **B**) Single point mutation (W389F) in S5-S6 linker plus three additional point mutations (R320N, L321A and R326I) in the S4 voltage sensor region of the Kv1.3-P93 channel. **C**) Single point mutation (Y447A) in the C-terminus of the Kv1.3-P121 channel.


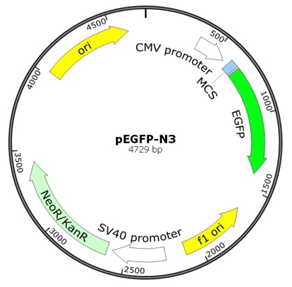

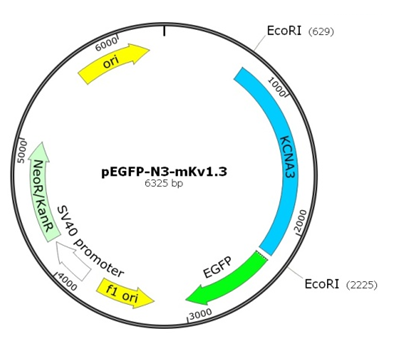

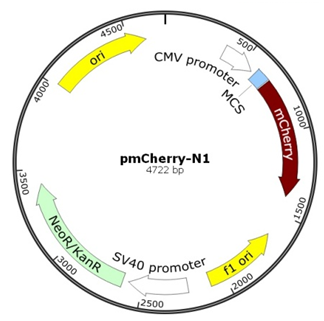

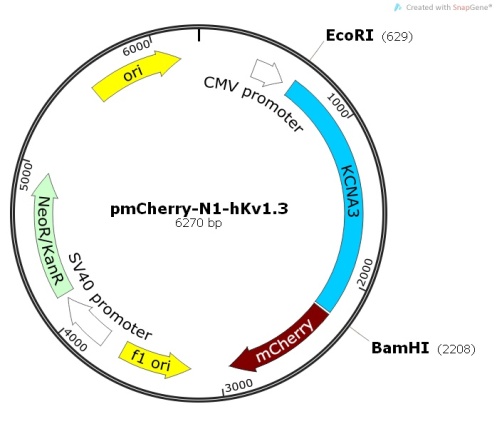


**A**

**B**

**C**

**D**

**Supplementary Figure 2. Plasmid maps for mutant Kv1.3 channels.** **A**) Original pEGP-N3 plasmid. The MCS region is between the CMV promoter and the EGFP. **B**) pEGP-N3 plasmid with the insertion of mKv1.3 (KCNA3) at the MCS region (pEGP-N3-mKv1.3). **C**) Original pmCherry-N1 plasmid with the MCS region between the CMV promoter and the mCherry. **D**) pmCherry-N1 plasmid with the insertion of hKv1.3 (KCNA3) at the MCS region (pmCherry-N1-hKv1.3).


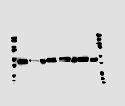


**HEK293/Kv1.3 –P93**

**HEK293/Kv1.3 –P118**

**HEK293/Kv1.3 –P89**

**HEK293/Kv1.3**

**50 kDa**

**Control HEK293**

**HEK293/Kv1.3 –P121**

**75 kDa**

**HEK293/Kv1.3 –P89**

**HEK293/Kv1.3 –P93**

**Supplementary Figure 3. Immunoblotting identifies stable expression of mutant Kv1.3 channels in HEK293 cells.** Western blot showing expression of Kv1.3 in control HEK293 cells and HEK293 cells with transgenic expression of Kv1.3 (HEK293/Kv1.3), Kv1.3-P118, Kv1.3-P121, Kv1.3 P-89 and Kv1.3-P93.

**A**

**B**

**Supplementary Figure 4. Nuclear Magnetic Resonance Spectroscopy demonstrating purity of synthesized PAPTP**. **A**) ^1^H – NMR spectra of synthesized PAPTP with resonances assigned to molecule structure. **B**) 13C-NMR spectra of synthesized PAPTP.

**Supplementary Figure 5. Increased Kv1.3 channel expression and K^+^ current in HEK293/Kv1.3 cells**. **A**) Representative images of control HEK293 (left) and HEK293/Kv1.3 cells (right) treated with Anti-Kv1.3 Clone L23/27, mouse monoclonal primary antibody and Alexa Fluor 488. Scale bars represent 10 µm. **B**) Representative Western blots for Kv1.3 protein (67-75kDa). **C**) Normalized Western blot densitometry quantifying Kv1.3 protein expression in both control HEK293 and HEK293/Kv1.3 cells (n = 5). **D**) Representative K^+^ ion currents in HEK293/Kv1.3 and control HEK293 cells, determined by whole-cell patch-clamp electrophysiology. **E**) Current-volatge (I/V) relationships of both HEK293/Kv1.3 cells (black circles) and control HEK293 cells (black squares). Currents evoked by step-depolarisations from -60 to +60mV. **F**) K^+^ currents (pA) in control HEK293 and HEK293/Kv1.3 cells evoked by a single step depolarization from −80 to +40 mV. Data are expressed as mean ± SEM (n = 6). Data analysed using Student’s t-test or Two Way ANOVA *p<0.05, ** p<0.01, ***p<0.001, ****p<0.0001.

**Control KV1.3**

**C**


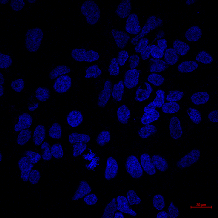

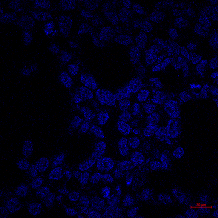


**A**

**B**

**ATP – B 46 KDa**

**ATP – B 46 KDa**

**Beta - Actin 43 KDa**

**Supplementary Figure 6. Kv1.3 channels localize to the mitochondria but do not increase cellular mitochondrial content**. MitoTracker controls in **A**) Control HEK293 and **B**) HEK293/Kv1.3 cells stained with diamidino-2-phenylindole (DAPI) (blue) for nuclei detection (excitation/emission wavelengths 360nm/460nm). Cells were excited at the MitoTracker detection wavelengths (579nm/599nm excitation/emission) in the absence of MitoTracker staining to control for autofluoresence. Scale bars represent 20 µm. **C**) Representative images of Western blots showing ATP synthase beta subunit protein (top row) and beta actin protein loading control (bottom row), in control HEK293 (left column) and HEK293/Kv1.3 (right column) cells.

**Supplementary Figure 7. PAPTP does not induce cell death.** Percentage viability of HEK293/Kv1.3 cells treated with PAPTP and assessed by trypan blue staining in the automated TC10 cell counter (n=4). Data are expressed as mean ± SEM.

**Supplementary Figure 8.** Effect of plasma membrane permeant mitochondrially-targeted Kv1.3 inhibitor PAPTP and non-organelle specific equivalent inhibitor PAP-1 on HEK293/Kv1.3 cell proliferation (n = 4). Data analysed using Student’s t-test, *p<0.05. Data are expressed as mean ± SEM.

**Supplementary Figure 9. A**) Proliferation of control HEK293 cells treated with 100 nM PAPTP expressed as a percentage increase in cell number over 3 days (n = 5). **B**) Residual O_2_ consumption (ROX) corrected Routine, Leak and maximal ETS Respiration in control HEK293 cells treated with PAPTP (100 nM; n = 5). Data are mean ± SEM.

**Supplementary Fig 10. Mitochondrial Kv1.3 channels regulate respiration independently of mitochondrial membrane potential, Ca^2+^ or NADH redox regulation. A**) Cairn Photometry measurement of TMRM fluorescence in control HEK293 and HEK293/Kv1.3 cells treated with 20 nM TMRM. Data is voltage-corrected ± SEM (n = 9). **B**) Flow cytometry measurement of TMRM initial fluorescence (20 nM) and following FCCP addition (20 µM) in control HEK293 cells (n = 7) and HEK293/Kv1.3 cells (n = 9). Data expressed as mean fluorescent intensity of TMRM fluorescence. **C**) The difference in TMRM (20 nM) fluorescence and TMRM fluorescence following treatment with FCCP (20 µM) in control HEK293 (n = 7) and HEK293/Kv1.3 (n = 9) cells to indicate differences in mitochondrial membrane potential. **D**) TMRM (20 nM) fluorescence in control HEK293 (n = 3) and HEK293/Kv1.3 cells (n = 6) ± PAPTP (100 nM). **E**) Rhod-2 AM Ca^2+^ dye fluorescence in control HEK293 cells (n = 5) and HEK293/Kv1.3 cells (n = 6) ± 20 µM FCCP to measure mitochondrial Ca^2+^. **F**) The difference in Rhod-2 AM fluorescence between baseline and FCCP treated control HEK293 and HEK293/Kv1.3 cells indicating mitochondrial Ca^2+^ (n = 5). **G**) NADH auto-fluorescence in control HEK293 cells (n = 5) or HEK293/Kv1.3 cells (n = 6) at baseline or following the addition of 20 µM FCCP or 0.5 µM rotenone and 2.5 µM antimycin. **H**) NADH range. The difference in NADH auto-fluorescence between the FCCP and dual Rotenone and Antimycin A treated control HEK293 cells (n = 5) or HEK293/Kv1.3 cells (n = 6). **I**) NADH autofluorescence in HEK293/Kv1.3 cells ± 100 nM PAPTP (n = 3). Data are ± SEM and analysed by either Students t-test or one-way ANOVA with Tukey’s multiple comparisons post hoc test,* P < 0.05, ** P < 0.01, *** P < 0.001, **** P < 0.0001. NS, not significant.

**Supplementary Table 1 Primer pairs for site directed mutagenesis of the Kv1.3 channel**. The base pairs from the mutated residues are highlighted in bold.

| Kv1.3 Channel | Mutation | Primer Sequence (5' to 3')  (forward and reverse) |  |
| --- | --- | --- | --- |
| Kv1.3-P89 | | W389F | 5'-tttaacagtatcccggatgccttc**ttc**tgggcagtagtaa-3'  5'-ttactactgccca**gaa**gaaggcatccgggatactgttaaa-3' |
| Kv1.3-P93 | | W389F  R320N  L321A  R326I | 5'-tttaacagtatcccggatgccttc**ttc**tgggcagtagtaa-3'  5'-ttactactgccca**gaa**gaaggcatccgggatactgttaaa-3  5'-tggccattctgagagtcatc**aac**gcagtaagggttttccgcatctt-3'  5'-aagatgcggaaaacccttactgc**gtt**gatgactctcaggatggccag-3'  5'-cctgagagtcatccgc**gca**gtaagggttttccgc -3'  5'-gcggaaaacccttac**tgc**gcggatgactctcagg -3'  5'-cgcctagtaagggttttc**atc**atcttcaagctctcccg-3'  5'-cgggagagcttgaagat**gat**gaaaacccttactaggcg -3’ |
| Kv1.3-P121 | | Y447A | 5’-AAT**GAATTC**CCGACATGACCGTGGTGCC-3’  5'- ATT**GGATCC**ACATCGGTGAATATCTTTTTGATG -3' |

**Supplementary Table 2. Plasmids for mutant Kv1.3 channels**. Plasmids sourced from Clontech. mKv1.3 (Source Bioscience), hKv1.3 (MagNA Pure Systems, Roche).

| **Mutant Kv1.3 Channel** | **Original Plasmid** | **Fusion Protein Plasmid** | **Primer Sequence (5' to 3')**  **(forward and reverse)** |
| --- | --- | --- | --- |
| Kv1.3-P89 and Kv1.3-P93 | pEGFP-N3 | pEGFP-N3-mKv1.3 | 5'-atc**gaattc**agacatgaccgtggtg-3'  5'-ca**gaattc**tggacatcagtgaatatcttcttga-3' |
| Kv1.3-P121 and Kv1.3-P118 (WT) | pmCherry-N1 | pmCherry-N1-hKv1.3 | 5’-AAT**GAATTC**CCGACATGACCGTGGTGCC-3’  5'- ATT**GGATCC**ACATCGGTGAATATCTTTTTGATG -3' |
